# BAIBA Involves in Hypoxic Training Induced Browning of White Adipose Tissue in Obese Rats

**DOI:** 10.3389/fphys.2022.882151

**Published:** 2022-06-23

**Authors:** Junpeng Feng, Xuebing Wang, Yingli Lu, Chang Yu, Xinyan Wang, Lianshi Feng

**Affiliations:** ^1^ Exercise Biology Research Center, China Institute of Sport Science, Beijing, China; ^2^ School of Sport Science, Beijing Sport University, Beijing, China; ^3^ College of Physical Education, Guangxi University, Nanning, China

**Keywords:** hypoxic training, obesity, BAIBA, lipid metabolism, browning

## Abstract

In recent years, obesity has become an important risk factor for human health; how to effectively prevent and reduce the occurrence of obesity is a hot research topic in recent years. Hypoxic training effectively improves abnormalities of lipid metabolism caused by obesity. The current study explored the effects of hypoxic training on BAIBA secretion and white fat browning in inguinal fat in obese rats. Analyses were performed by HPLC/MS/MS—MS/MS, RT-q PCR and western blot methods. The findings showed that 4 weeks of hypoxic training reduced body weight, Lee’s index, and regulated blood lipid profile in obese rats. Hypoxic training up-regulated BAIBA concentration in gastrocnemius muscle and circulation in obese rats. Hypoxic training significantly upregulated expression of PPARα and UCP-1 in inguinal fat of obese rats and increased white fat browning. The findings showed that BAIBA may involve in improveing blood lipid profile and white fat browning by modulating PPARα and UCP-1 expression.

## 1 Introduction

Obesity is a major public health concern. The global obesity prevalence has increased by approximately 1.5-fold for adults and 2-fold for children in the past 20 years (WHO, 2020). China CDC reported that the national overweight rate for adults aged 18 and above was 30.1% in 2015 and the prevalence of obesity rate was 11.9% (Wang et al., 2017), and is currently on the rise. Studies are currently, exploring effective methods for preventing and reducing occurrence of obesity.

Mammals have two main types of adipose tissue including the white adipose tissue (WAT) and brown adipose tissue (BAT) (Mueller, 2016; Jeremic et al., 2017). White adipose tissue originates from Myf5-progenitor cells, which are characterized by accumulation of single-compartment lipid droplets and few mitochondria. The main function of white adipose tissue is energy storage. Brown adipocytes originate from Myf5+ progenitor cells and are characterized by multicompartmental lipid droplets containing abundant mitochondria. The main function of brown adipocytes is to maintain body temperature though non-shivering thermogenesis (Wu et al., 2012). The inner mitochondrial membrane of brown adipocytes is rich in uncoupling protein 1 (UCP-1), which causes uncoupling of electron transfer and ATP production processes of mitochondrial oxidative respiration. Uncoupling of these two processes results in emission of large amounts of energy in form of heat and energy consumption, however, this thermogenic effect is highly regulated in the body (Rodriguez et al., 2020).

There is a third type of adipocytes similar BAT called beige/brite adipocyte occurs in WAT. Beige adipocytes are differentiated from WAT under induced conditions. Beige cells have similar thermogenic capacity as brown adipocytes under fully activated conditions. Moreover, beige adipocytes have a similar origin as WAT but have unique marker genes such as Transmembrane protein 26 (TMEM-26), CD137 and T-box 1 (Tbx1) (Dempersmier and Sul, 2015; Mueller, 2016).

Cold exposure and fasting induces production of beige fat in WAT through a process known as white fat browning (Poher et al., 2015). An important hallmark of white fat browning is increased expression of UCP-1 and increased thermogenesis. The classical pathway of white fat browning is association of PR domain-containing 16 (PRDM16) with CCAAT-enhancer binding proteins-β (C/EBP-β) through its zinc finger domain. This association forms a transcriptional complex that induces peroxisome proliferator-activated receptor-α/γ (PPARα/γ) and peroxisome proliferator-activated receptor-γ coactivator 1-α (PGC1-α) expression. Peroxisome proliferator activated receptor-γ coactivator1-α (PRDM16 binds to PPAR-γ and PGC-1α to form a complex which induces UCP-1 expression and browning (Kim et al., 2016; Jeremic et al., 2017).

Exercise is a good method for weight loss. Regular exercise increases energy expenditure of the body, reduces body fat, and improves body composition (Wang et al., 2019). Expression levels of beige adipocyte markers (such UCP-1 and PRDM16) in white fat, mainly in subcutaneous fat are upregulated by different exercise modalities (such as free spinning wheel, running platform and swimming) and different intervention durations (one-time or long-term), indicating that regular exercise promotes rodents white fat browning (Gorgens et al., 2015; Rocha-Rodrigues et al., 2016).

The mechanism of exercise-induced white fat browning may be regulated by other pathways besides activation of the sympathetic nervous system. In addition to consumption of a large amount of energy during exercise, skeletal muscle secretes several myokines to regulate metabolic disorders caused by obesity (Gorgens et al., 2015; Hoffmann and Weigert, 2017). A previous study reported that exercising muscles secrete interleukin-6 (IL-6) which is released into the blood (Steensberg et al., 2000). Studies are currently exploring the muscle as an endocrine organ, and cytokines secreted and released by muscles are called “myokines.” Some of the myokines released by muscles includeIL-6 (Steensberg et al., 2000), fibroblast growth factor 2 (FGF21) (Fisher et al., 2012), brain-derived neurotrophic factor (BDNF), irisin (BDNF), and myokine (Irisin) (Bostrom et al., 2012). These muscle factors can act on the liver and adipose tissue to regulate glucolipid metabolism (Hoffmann and Weigert, 2017; Castillo-Armengol et al., 2019; Kirk et al., 2020).

β-Aminoisobutyric acid (BAIBA) has been reported recently as a newly discovered muscle factor (Kammoun and Febbraio, 2014; Roberts et al., 2014). BAIBA is a natural metabolite of thymine and valine secreted by myocytes during exercise. BAIBA exists as two main enantiomeric isomers in organisms, D-BAIBA and L-BAIBA. D- BAIBA is produced by the cytosolic thymine metabolic pathway, which mainly involves dihydropyrimidine dehydrogenase (DPYD), dihydropyrimidinase (DPYS) and β-ureidopropionase (β-ureidopropionase enzymes). L-BAIBA is produced through catabolism of the branched-chain amino acid, L-valine through transamination of L-methyl-malonyl semialdehyde (L-MMS), a downstream product of L-valine, and L-glutamic acid by the action of mitochondrial 4-aminobutyric acid transaminase enzyme (Tanianskii et al., 2019). BAIBA reduces body fat and its activity is not dependent on changes in energy intake or physical activity levels, but rather on changes in basal oxygen consumption. BAIBA can promote white adipose tissue thermogenesis through adrenaline-dependent and-independent pathways andwhite fat browning (Kitase et al., 2018). In addition, exercise increases plasma BAIBA levels and plasma BAIBA levels are negatively correlated with the risk of metabolic diseases (Kammoun and Febbraio, 2014; Roberts et al., 2014).

Previous studies conducted by our group reported that Hypoxic Training is effective in regulating lipid metabolism in obese rats. The findings showed that the main mechanisms involve skeletal muscle fatty acid metabolism and liver lipid metabolism (Lu et al., 2014; Lu et al., 2016; Gao et al., 2020). However, effect of Hypoxic exercise on BAIBA secretion in obese rats has not been fully elucidated. Moreover, studies have not fully explored whether BAIBA is involved in regulation of lipid metabolism and white fat browning induced by Hypoxic exercise. In the study, a hypoxic exercise model was established in obese rats. In addition, mechanisms of the effects of hypoxic exercise on lipid metabolism and white fat browning in obese rats were explored by determining the levels of BAIBA.

## Materials and Methods

### Animals and Experimental Design

A total of 110 3-week-old male SD rats were used to establish an obesity model in the current study. Animals were housed in separate cages, five rats per cage, under a light/dark cycle of 12 h/12 h, a temperature of 22 ± 1°C and 40%–60% humidity. Animals were fed with normal chow feeding for a week, then randomly assigned to two groups. The first group comprised 20 rats fed with normal chow and 90 rats fed with high-fat chow diet *ad libitum*. Through our previous research, we found that the success rate of obesity model modeling was about 60%, so 90 animals were used to establish theobesity model. The feed composition was as follows: normal feed diet comprising experimental rat growth maintenance pellet feed (3.40 kcal/g, 65% of energy from carbohydrate, 12% of energy from fat); high-fat feed comprising high-fat feed from Research Diets, United States (item no. D12451, 4.73 kcal/g, 35% of energy from carbohydrate, 45% of energy from fat). Animals were fed for 12 weeks then 32 obese rats were selected from the high-fat diet group based on their body weight and Lee’s index.

The calculation formula of Lee’s index:

$$\text{Lee’s index =}\frac{\sqrt[{3}]{body\;weight\left(g\right)}}{body\;length\left(cm\right)}\text{∗}10^{3}$$

During the exercise and hypoxia intervention, each group was guaranteed to have no less than 10 rats. However, due to factors such as injury during the training process, the number of rats in each group included in this study for statistical analysis was eight in each group. The standard group (NC group) and the standard exercise group (NE group) were selected from the normal diet group, with eight rats in each group. Obese rats were randomly assigned to obese normoxic control group (ONC group), obese normoxic exercise group (ONE group), obese hypoxic control group (OHC group), and obese hypoxic exercise group (OHE group), with eight rats in each group. Rats in each group had similar body weight and training condition. Exercise intervention was performed in the NE, ONE, and OHE groups; hypoxic intervention was performed in the OHC and OHE groups. The duration of intervention was 4 weeks in all groups. The experiments were reviewed and approved by the ethical committee on treatment and handling of experimental animals.

### Exercise Intervention Programs

All groups underwent 2 weeks of running table adaptation training. The training speed was increased from 16 m/min to 25 m/min and the exercise time was increased from 20 min/d to 60 min/d during the 2 weeks. Endurance training was conducted using a horizontal animal running platform with a running speed of 20 m/min in the OHE group and 25 m/min in the ONE group for 1 h/days, 5 days/weeks and the training lasted for 4 weeks. The previous experiments showed that the blood lactate concentration of the rats in the OHE group and the ONE group was basically the same during exercise, that is, the exercise intensity of the HE group and the NE group was the same (He et al., 2012).

### Hypoxia Intervention Program

Hypoxia generator (GA15FF-13 twin-screw air compressor and CA-200AT nitrogen generator) purchased from Tianjin Senro Technology Co., Ltd. was used to create an atmospheric pressure hypoxic experimental environment. OHC and OHE groups were subjected to a hypoxic intervention by simulating living and/or training in an altitude of 3,500 m (oxygen concentration of 13.6%) for a period of 4 weeks.

### Sample Collection

Sampling was performed after 48 h after the end of the last exercise to minimize effect of acute exercise on the relevant indexes. Rats were weighed and the body length determined, then 10% trichloroacetaldehyde hydrate for intraperitoneal anesthesia was administered intraperitoneally to induce anesthesia at a dose of 0.3 ml/100 g body weight. After induction of anesthesia, blood was drawn from the abdominal aorta and centrifuged for subsequent analysis. Inguinal fat and gastrocnemius muscle were sectioned on ice, divided in two portions and immediately wrapped with numbered tin foil. Samples were placed in liquid nitrogen, and later transferred to −80° ultra-low temperature refrigerator for further use. Blood lipid indexes were analyzed using a Hitachi 7,600 automatic biochemical analyzer following the manufacturer’s instructions.

### Real Time qPCR

Relative expression levels of PPAR α and UCP-1 mRNA in inguinal fat were determined by RT-qPCR.

Total inguinal fat RNA was extracted using TriZol reagent (Invitrogen, United States) according to the manufacturer’s instructions. Purity of total RNA was determined by agarose gel electrophoresis and images were obtained with EUV-LDUV gel imaging system (KoreaBiotech, Korea).

cDNA synthesis and RT-qPCR assays were performed using M-MLV reverse transcriptase and Premix Taq™ (Ex Taq™ Version 2.0), which were purchased from Bao Biological Engineering (Dalian) Co. Primers were synthesized by Beijing Tianyi Huiyuan Biotechnology Co., Ltd. β-Actin was used as the housekeeping gene. The primer sequences used are presented in Table 1. ddCT method was used to determine the relative expression of target genes.

**TABLE 1:** Real-time PCR Primer sequences.

Primer Name	Primer Sequence	Product Length/Bp
*UCP-*1-F	TCC​CTC​AGG​ATT​GGC​CTC​TAC	101
*UCP-*1-R	GTC​ATC​AAG​CCA​GCC​GAG​AT	
*PPAR-α*-F	TCC​ACG​AAG​CCT​ACC​TGA​AGA​ACT	187
*PPAR* -*α*-R	AAT​CGG​ACC​TCT​GCC​TCC​TTG​TT	
*β-Actin*-F	GAA​GTG​TGA​CGT​TGA​CAT​CCG	282
*β-Actin*-R	GCC​TAG​AAG​CAT​TTG​CGG​TG	

### Western Blot Analysis

Protein expression levels of PGC-1 α, PPAR α, and UCP-1 in inguinal fat samples were determined by western blot.

Total inguinal fat protein was extracted using tissue protein lysate. Protein concentration was determined using BCA protein quantification kit according to the manufacturer’s instructions. The required sampling volume for each sample was determined based on the protein concentration and the samples were mixed with 2 * loading buffer to obtain 50 µg of total protein for each sample.

10% separation gel was prepared and gel electrophoresis of the samples was performed. The electrophoresis voltage was 120 V and electrophoresis was terminated when bromophenol blue electrophoresis reached the bottom of the gel. After electrophoresis, samples were transferred to PVDF membrane at a constant current of 300 mA for 1 h. Membranes were blocked with 5% skimmed milk powder prepared with TBST. The membrane was then immersed and slowly shaken on a shaker and at room temperature for 60 min. Membranes were incubated with primary antibody (see Table 2 for the primary antibody dilution ratio) overnight at 4°C. Samples were washed thrice with western wash solution under slow shaking on a side-swinging shaker for 5–10 min. Horseradish peroxidase (HRP)-labeled secondary antibody was diluted with western secondary antibody diluent at a ratio of 1:10,000. The membrane was incubated with the diluted secondary antibody for 1 h at room temperature on a side-swinging shaker with slow shaking. Membranes were washed using western wash solution on a shaker under slow shaking thrice for 5–10 min. Liquid A: liquid B of the ECL luminescence reagent was prepared based on the size of the membrane in a 1:1 ratio by volume. The ECL was evenly placed on the PVDF film, then the X-ray film was obtained film strips of the same size were obtained. The X-ray film was then placed directly on top of the film. When the strips were clear enough, they were rinsed in water, and the X-ray film was placed into the fixing solution. The developed negative was scanned and the image was analyzed in grayscale using IPP6 software. Statistical analysis was performed on the obtained grayscale values. β-Actin was used as an internal control and the results were expressed as protein/internal reference protein expression levels.

**TABLE 2:** Dilution ratio of primary antibodies.

Primary antibody	Antibody Manufacturers	Catalog No.	Dilution ratio
PPAR-α	Abcam	ab24509	1:3,000
PGC-1 α	Abcam	ab54481	1:1,000
UCP-1	Abcam	ab23841	1:1,000
β-Actin	CST	4,967	1:1,000

### HPLC/MS/MS Analysis

Samples were weighed and homogenized with distilled water. The homogenate was centrifuged at 13,200 rpm for 1 min, and the supernatant was obtained for subsequent analysis. 400 µl of protein precipitant (including internal standard) was added to 100 µl of the supernatant and the mixture was vortexed for 1 min. The sample was the let to stand for 5 min, then centrifuged at 13,200 rpm for 4 min, and the supernatant was obtained for subsequent analysis.

Blood was drawn from the abdominal aorta, and serum was obtained by centrifugation at 3,000 rpm for 15 min. The supernatant was further centrifuged at 13,200 rpm for 4 min.

Concentration of BAIBA in the samples was determined by HPLC-MS/MS method. Thee LC liquid phase was an Ultimate 3,000 high performance liquid chromatograph (DIONEX, United States) and the MS mass spectrometer was an API 3200 Q TRAP liquid mass spectrometer (AB, United States). The standards, methanol and nitrile were of analytical purity (Fisher, United States).

### Statistical Analysis

Statistical analysis was conducted using SPSS (United States). Data were presented as mean ± SD and differences among groups were compared by ANOVA. Correlation analysis was performed using bivariate correlation analysis, with R ≥ 0.8 being highly correlated, 0.8 > R > 0.3 being moderately correlated, and R ≤ 0.3 being lowly correlated. A *p* < 0.05 was considered statistically significant.

## Results

### Effects of Normoxia Exercise and Hypoxia Exercise on Body Weight and LEE’s Index in Rats

**FIGURE 1:**
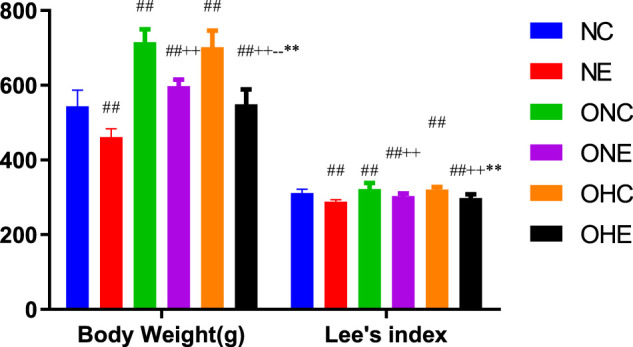
Changes in Body Weight and Lee’s Index of Rats after Normoxic and Hypoxic Exercise. *n* = 8, ^#^ *p* < 0.05 vs. NC group; ##*p* < 0.01 vs. NC group; +*p* < 0.05 vs. ONC group; ++*p* < 0.01 vs. ONC group; -*p*<0.05 vs. ONE group; --*p*<0.01 vs. ONE group; *p < 0.05 vs. OHC group; **p < 0.01 vs. OHC group; following tables same.

The findings showed that the body weight of rats in the ONC and OHC groups was significantly higher compared with that of rats in the NC group (Figure 1). Notably, the body weight of rats in the ONE and OHE groups was significantly lower compared with that of rats in the ONC group (*p* < 0.01). Analysis showed that the body weight of rats in the OHC group was not significantly different from that of rats in the ONC group (*p* > 0.05). However, the body weight of rats in the OHE group was significantly lower compared with that of rats in the ONE group (*p* < 0.01). These findings indicate that normoxic and hypoxic exercise reduces body weight of obese rats. Lee’s index of rats in ONE and OHE groups was significantly lower compared with that of rats in the ONC group (*p* < 0.01). Lee’s index of rats in OHC group was not significantly different from that of rats in the ONC group (*p* > 0.05). These findings indicate that normoxic and hypoxic exercise decreases the Lee’s index in obese rats.

### Effects of Normoxia Exercise and Hypoxia Exercise on Levels of Blood Lipids in Rats

The findings showed that level of TG of rats in the ONC group was significantly higher compared with that in the NC group (*p* < 0.01, Table 3). In addition, LDL level was significantly lower compared with that in the NC group (*p* < 0.01, Table 3). Levels of TC, TG, and LDL of rats in the ONE group were significantly lower compared with the levels in the ONC group (*p* < 0.01). However, analysis showed no significant difference in HDL levels between the ONE group and ONC group (*p* > 0.05). Levels of TC, TG, HDL, and LDL of rats in the OHC group were not significantly different from the levels in the ONC group (*p* > 0.05). The finding showed that levels of TC, TG and LDL of rats in the OHE group were significantly lower compared with the levels in the HC and NC groups (*p* < 0.01, *p* < 0.01). Notably, HDL level of rats in the OHE group was significantly higher compared with the level of HDL in the OHC and ONC groups (*p* < 0.01, *p* < 0.01). Analysis showed that the level of LDL of rats in the OHE group was significantly lower compared with level in rats in the ONE group (*p* < 0.01).

**TABLE 3:** Changes in Blood Lipid levels in Rats after Normoxic and Hypoxic Exercise (*n* = 8).

	NC	NE	ONC	ONE	OHC	OHE
TC (mmol/L)	1.23 ± 0.25	0.88 ± 0.21^##^	1.41 ± 0.17	0.97 ± 0.12^++##^	1.30 ± 0.10	0.86 ± 0.23^++**##^
TG (mmol/L)	0.27 ± 0.04	0.17 ± 0.03^#^	0.47 ± 0.17^##^	0.31 ± 0.03^++^	0.41 ± 0.11^##^	0.25 ± 0.08^++**^
HDL (mmol/L)	0.38 ± 0.04	0.41 ± 0.10	0.33 ± 0.05	0.38 ± 0.06	0.35 ± 0.05	0.46 ± 0.13^++**^
LDL (mmol/L)	0.37 ± 0.10	0.30 ± 0.06^#^	0.31 ± 0.02^#^	0.27 ± 0.02^++^	0.28 ± 0.05	0.23 ± 0.03^##++--**^

n = 8, ^#^p < 0.05 vs. NC group; ^##^p < 0.01 vs. NC group; +p < 0.05 vs. ONC group; ++p < 0.01 vs. ONC group; -p < 0.05 vs. ONE group; --p < 0.01 vs. ONE group; *p < 0.05 vs. OHC group; **p < 0.01 vs. OHC group; following tables same.

### Effects of Normoxia Exercise and Hypoxia Exercise on mRNA Expression Levels of PGC-1α, PPAR α and UCP-1 in Inguinal Fat of Rats

**FIGURE 2:**
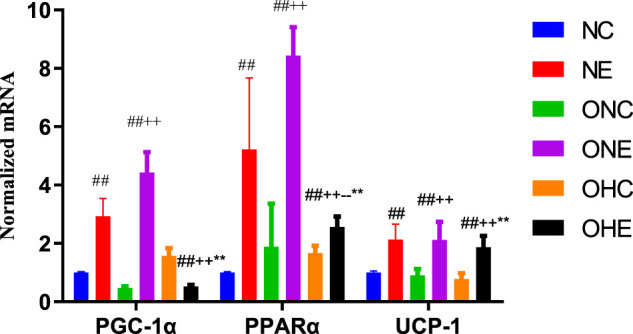
mRNA expression levels of PGC-1α, PPAR α, and UCP-1 in inguinal fat of rats. mRNA expression levels of PPARα and UCP-1 in inguinal adipose of rats of NC, NE, ONC, ONE, OHC, and OHE groups were determined using β-Actin as a reference gene. *n* = 8, ^#^ *p* < 0.05 vs. NC group; ^##^ *p* < 0.01 vs. NC group; +*p* < 0.05 vs. ONC group; ++*p* < 0.01 vs. ONC group; -*p* < 0.05 vs. ONE group; --*p* < 0.01 vs. ONE group; *p < 0.05 vs. OHC group; **p < 0.01 vs. OHC group; following tables same.

The findings showed that the mRNA expression levels of *PGC-1α, PPAR α*, and *UCP-1* in the inguinal fat of rats in the NE group were significantly higher compared with the levels in rats in the NC group (*p* < 0.01, Figure 2). In addition, mRNA expression levels of *PGC-1α, PPAR α*, and *UCP-1* in the inguinal fat of rats in the ONE group were significantly higher compared with the levels in rats in the ONC group (*p* < 0.01). Analysis showed that the mRNA expression levels of *PPAR α* and *UCP-1* in the inguinal fat of rats in the OHE group were significantly higher compared with the levels in rats in the ONC and OHC groups (*p* < 0.01, *p* < 0.01, *p* < 0.01).

### Effects of Normoxia Exercise and Hypoxia Exercise on Protein Expression Levels of PGC-1α, PPAR α and UCP-1 in the Inguinal Fat of Rats

**FIGURE 3:**
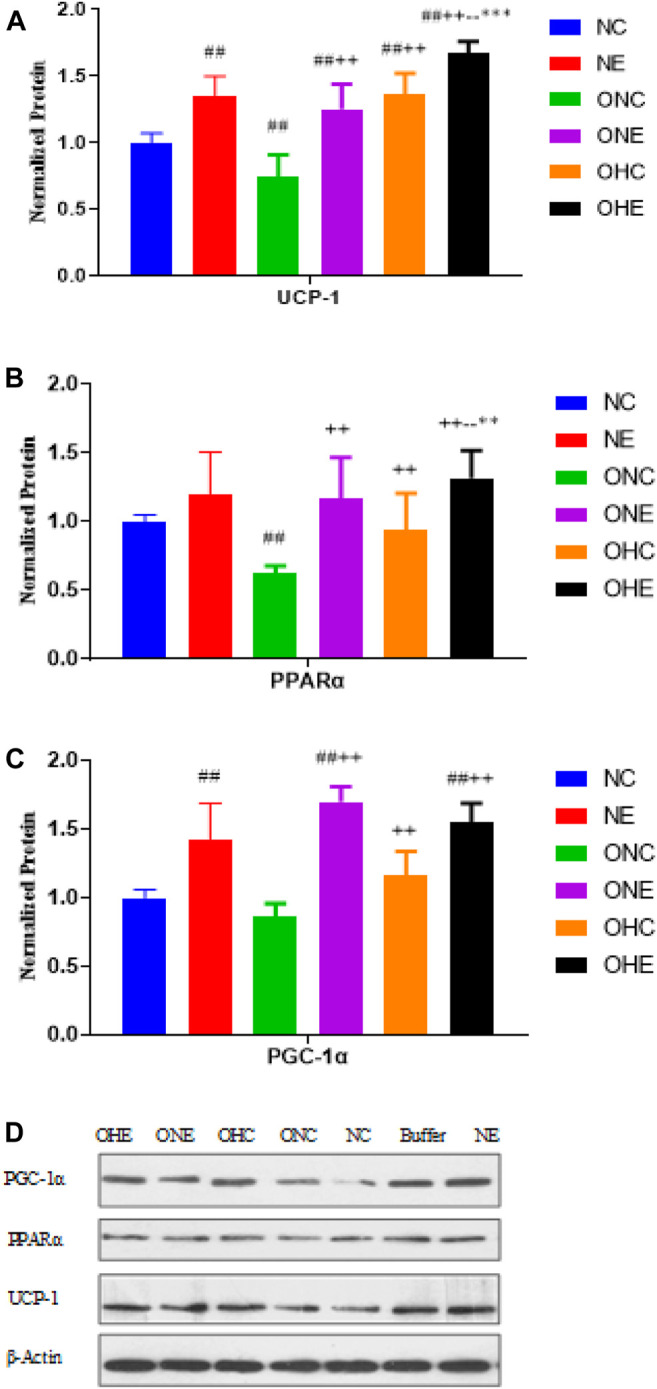
Protein expression levels of UCP-1 **(A)**, PPAR α **(B)** and PGC-1α **(C)** in inguinal adipose of rats after normoxic and hypoxic exercise. Protein expression levels of PPARα and UCP-1 in inguinal adipose of rats in NC, NE, ONC, ONE, OHC, and OHE groups were expressed as relative levels based on the expression level of β-Actin **(D)**. *n* = 8, ^#^ *p* < 0.05 vs. NC group; ^##^ *p* < 0.01 vs. NC group; +*p* < 0.05 vs. ONC group; ++*p* < 0.01 vs. ONC group; -*p* < 0.05 vs. ONE group; --*p* < 0.01 vs. ONE group; *p < 0.05 vs. OHC group; **p < 0.01 vs. OHC group; following tables same.

The findings showed that the protein expression levels of PGC-1α and UCP-1 in the inguinal fat of rats in the NE group were significantly higher compared with protein levels in rats in the NC group (*p* < 0.01, Figure 3). Protein expression levels of PGC-1α, PPAR α, and UCP-1 in the inguinal fat of rats in the ONE group were significantly higher compared with the protein levels in rats in the ONC group (*p* < 0.01). Protein expression levels of PGC-1α, PPAR α, and UCP-1 in the inguinal fat of rats in the OHC group were significantly higher compared with the protein levels in rats in the ONC group (*p* < 0.01). Protein expression levels of PPAR α and UCP-1 in the inguinal fat of rats in the OHE group were significantly higher compared with the protein levels in rats in the ONC OHC groups (*p* < 0.01, *p* < 0.01, *p* < 0.01). Moreover, the protein expression level of PGC-1α in the OHE group was significantly higher compared with the protein level in rats in the ONC group (*p* < 0.01).

### Effects of Normoxic Exercise and Hypoxic Exercise on Expression Level of BAIBA in Gastrocnemius Muscle and Blood of Rats

The findings showed that the levels of BAIBA in the gastrocnemius muscle and blood of rats in the NE group were significantly higher compared with the levels in the NC group (*p* < 0.01, Table 4). In addition, level of BAIBA in gastrocnemius muscle of rats in the ONE group was significantly higher compared with the level in rats in the ONC group (*p* < 0.01). However, analysis showed no significant difference between BAIBA level in the blood of rats in the OHC group and that in the ONE group (*p* > 0.05). Level of BAIBA in gastrocnemius muscle of rats in the OHE group was significantly higher compared with the level in rats in the ONC and OHC groups (*p* < 0.01, *p* < 0.01). Moreover, level of BAIBA in the blood of rats in the OHE group was significantly higher compared with the level in rats in the OHC group (*p* < 0.01). However, analysis showed no significant difference in the level of BAIBA in rats in the ONC and ONE groups (*p* > 0.05, *p* > 0.05).

**TABLE 4:** Levels of BAIBA in Gastrocnemius Muscle and Blood of Rats after normoxic and hypoxic exercise (*n* = 8).

	NC	NE	ONC	ONE	OHC	OHE
Muscle BAIBA(ng/mg)	1.12 ± 0.04	6.47 ± 1.24##	1.68 ± 1.02	7.07 ± 2.50 ++##	2.89 ± 1.13	3.72 ± 1.12++--
Blood BAIBA(ng/mL)	23.28 ± 7.50	37.94 ± 8.49##	35.56 ± 12.17	44.28 ± 22.17##	14.09 ± 2.72++	39.15 ± 16.89**

n = 8, #p < 0.05 vs. NC group; ##p < 0.01 vs. NC group; +p < 0.05 vs. ONC group; ++p < 0.01 vs. ONC group; -p < 0.05 vs. ONE group; --p < 0.01 vs. ONE group; *p < 0.05 vs. OHC group; **p < 0.01 vs. OHC group; following tables same.

### Relationship Between BAIBA Level, and Blood Lipid Level and Browning Index in Rats

The findings showed that BAIBA level in rat gastrocnemius muscle was negatively correlated with rat TC level and positively correlated with PPARα and UCP-1 mRNA expression levels in rat inguinal fat (Table 5). Moreover, blood BAIBA level was negatively correlated with rat TC level and positively correlated with PPARα and UCP-1 mRNA expression level in rat inguinal fat (*p* < 0.05, *p* < 0.01).

**TABLE 5:** Relationship between BAIBA and blood lipid, and browning index in Rats (n = 8).

		Muscle BAIBA	Blood BAIBA
		Correlation Value (*p* Value)	Correlation Value (*p* Value)
TC	Pearson correlation	−0.478 (0.006)%%	−0.422 (0.016) %
TG		−0.313 (0.082)	−0.277 (0.124)
HDL		0.167 (0.360)	0.139 (0.448)
LDL		−0.338 (0.059)	−0.194 (0.288)
PPARα mRNA		0.791 (0.000) %%	0.355 (0.046) %
UCP-1 mRNA		0.568 (0.001) %%	0.550 (0.001) %%
PGC-1α protein		0.372 (0.187)	0.164 (0.273)
PPARα protein		0.425 (0.015) %	0.339 (0.058)
UCP-1 protein		0.246 (0.175)	0.279 (0.122)

%significant difference p < 0.05, %%significant difference p < 0.01.

## Discussion

The findings of the current study showed that hypoxic exercise and normoxic exercise significantly reduced body weight and Lee’s index in obese rats (*p* < 0.01). In addition, hypoxic exercise had a significantly higher effect in reduction of body weight in obese rats was more effective in improving Lee’s index compared with the effect of exercise under normoxia. These findings indicate that hypoxic exercise intervention in is more effective in weight reduction in obese rats compared with exercise under normal conditions.

Moreover, hypoxic exercise significantly improved lipid metabolism. Obese people present with significantly higher TC, TG and LDL levels and significantly lower HDL levels compared with healthy individuals. Abnormal lipid metabolism leads to changes in lipid parameters, which is an important risk factor for obesity-induced cardiovascular diseases. However, in this study, the HDL and LDL of the ONC group even tended to be lower than those of the NC group, and further research is needed. Exercise can improve lipid metabolism in the body, as indicated by decrease in TC, TG, and LDL levels and increase in HDL levels in rats subjected to exercise. Notably, high-intensity interval training induced significantly higher effects on levels of lipid parameters compared with the effect of moderate-intensity aerobic training. Acute hypoxic exposure decreases the levels of triglycerides with 50 and 48 carbons, whereas TGs containing 48-50 carbons are mainly associated with adipogenesis (Kennedy et al., 2001). Studies report that acute hypoxic exposure leads to increase in free fatty acid levels in the blood resulting in increased fatty acid metabolism. Long-term hypoxic exposure (6 weeks or 30 days) causes significant decrease in TG, however, the trends in TC, HDL, and LDL levels are not correlated with the duration of hypoxic exposure and/or degree of hypoxia (Kennedy et al., 2001; Siques et al., 2014; Song et al., 2020). The findings of the current study indicated that normoxic and hypoxic exercise reduced TC, TG, and LDL levels in obese rats. However, the effect of hypoxic exercise in improving HDL and LDL was significantly higher compared with the effect of normoxic exercise. Du et al. (2020) reported consistent findings that increase in altitude was correlated with decrease in TG levels and the decrease was highly correlated hypoxic exposure, however, the effects on TC, LDL, and HDL levels were not correlated with degree of hypoxic exposure.

The preliminary research of our research group shows that 4 weeks of hypoxic training can be achieved by increasing Sterol Regulatory Element Binding protein-1C (SrebP-1C), acetyl-Coa Carboxylase 1 (ACC1) and Fatty Acid in the liver The expression level of Synthetase (FASN) and the expression level of Carnitine Palmitoyl Transferase 1A (CPT1A) in liver can be down-regulated to improve liver lipid metabolism by inhibiting fatty acid synthesis in liver, increasing fatty acid transfer and oxidation in liver, and thus improving body lipid metabolism. (Jing Wen, 2018).

Oxygen concentration affects adipocyte function. Exposure of white adipocytes to hypoxic conditions in culture leads to changes in the expression of over 1,000 genes. The secretion of several adipokines associated with inflammation is upregulated by hypoxia and shifts from oxidative metabolism to anaerobic glycolysis. Glucose utilization is increased in hypoxic adipocytes with a corresponding increase in lactate production. Importantly, hypoxia induces insulin resistance in adipocytes and leads to the development of adipose tissue fibrosis (Trayhurn, 2013; Gozal et al., 2017).

Exercise also induces functional changes in adipose tissue. In particular, after Bostrom et al. (2012) showed in 2012 that irisin as a myokine can promote white fat browning, more and more studies in recent years have demonstrated that exercise can promote white fat browning, especially the promotion effect of aerobic exercise is more obvious (Rocha-Rodrigues et al., 2016; Rodriguez et al., 2017).

Hypoxic training can promote the expression of UCP-1 through various mechanisms, including the activation of AMPK signaling pathway *in vivo* by hypoxic exercise, which on the one hand can inhibit AMPK levels in hypothalamus, resulting in decreased appetite and downregulation of body fat, and on the other hand, AMPK can stimulate increased expression of PGC-1α mRNA and protein, thus inducing the conversion of white fat to brown fat. However, on the other hand, it has also been shown that hypoxia downregulates neuropeptide Y (NPY) expression levels and that NPY gradually decreases with increasing altitude and duration of hypoxia. In contrast, NPY may inhibit UCP-1 expression by specifically expressing Y5R on the surface of BAT(Kotz et al., 2000). It indicates that hypoxia can affect UCP-1 expression by affecting NPY. The effect of hypoxia on UCP-1 is still unclear and needs to be explored in further studies.

The current study explored the effects of hypoxic exercise on the expression levels of PPARα and UCP-1 in white fat of obese rats and the correlation od these levels with BAIBA level. The findings showed that both normoxic and hypoxic exercise upregulated expression of *PPAR α* mRNA in inguinal fat of rats. Notably, upregulation of *PPAR α* mRNA expression was higher under normoxic exercise compared with that under hypoxic exercise. In addition, hypoxic and normoxic exercise upregulated *UCP-1* mRNA expression in rat inguinal fat, and the finding showed no significant difference between the two forms of exercise on upregulation of *UCP-1* mRNA expression. Normoxic and hypoxic exercise upregulated PPAR α and UCP-1 protein expression in inguinal fat of obese rats, with a higher effect observed under hypoxic exercise. Moreover, hypoxic environment upregulated PPAR α protein expression in inguinal fat of obese rats. In this study, the mRNA expression trends of PGC-1α and other indicators are not completely consistent with the protein expression trends, which may be due to the fact that PGC-1α has a co-activation effect with PPAR-γ and PRDM-16 during browning, mainly at the mRNA level. In this study, we found that hypoxic exercise had an advantage in promoting UCP-1 protein expression, but hypoxic training did not seem to have an advantage over normoxic training in promoting the expression of PPARα and PGC-1α. This may involve appetite suppression and enhanced hepatic lipid metabolism induced by hypoxia and hypoxic training. On the other hand, this study selected the most favorable oxygen concentration for improving lipid metabolism, which may not be the optimal oxygen concentration for promoting browning, and the exact mechanism needs to be further investigated.

PPARα regulates gene transcription by acting on the promoter of target genes, regulates lipid metabolism, suppresses inflammatory responses, and promotes white fat browning. Hypoxic exposure, hypoxic exercise, cold exposure and starvation can affect PPARα expression. Studies in mice exposed to hypoxia found elevated ACC expression in the liver, increased lipid synthesis in the liver, and significantly lower PPARα mRNA expression and reduced fatty acid oxidation. Subsequently, it was found that hypoxic exercise decreased CPT1 protein expression, inhibited the transport of long-chain fatty acids into mitochondria, and increased hepatic fatty acid transport, thereby reducing body weight and body fat in rats (Tong, 2005; Piguet et al., 2010; Urdampilleta et al., 2012). In this study, we found that both hypoxic and low aerobic exercise upregulated PPARα mRNA expression in adipose tissue, and exercise had a more significant effect on promoting PPARα mRNA.

BAIBA is a non-protein amino acid (Tanianskii et al., 2019) and exercise significantly affects BAIBA secretion. Notably, BAIBA is mainly produced and secreted during muscle contraction. Stautemas et al. (2019) reported that the concentration of D-BAIBA in blood under resting state was approximately 67-fold higher compared with levels of L-BAIBA (1734 ± 821 nM vs. 29.3 ± 7.8 nM). In addition, D-BAIBA increased by approximately 13% and L-BAIBA increased by approximately 20% after 1 h of cycling at maximum output power intensity. The two conformations of BAIBA play roles as muscle factors (Stautemas et al., 2019). A study by Roberts et al. reported that BAIBA concentrations in gastrocnemius muscle of wild-type mice increased by approximately 5.2-fold (*p* < 0.01) after 3 weeks of free spinning exercise, and blood BAIBA levels increased by approximately 19% (*p* < 0.01) (Roberts et al., 2014).

In this study, we found Hypoxic exercise also led to an increase in BAIBA secretion in the gastrocnemius muscle, but the increase was lower than that of normoxic exercise; hypoxic exercise could ameliorate the decrease in blood BAIBA concentration caused by the hypoxic environment and restore it to a level exceeding normoxic rest. Previous studies have shown that staying at high altitude results in increased metabolism of BCAA, BCAA catabolism in skeletal muscle begins with the transamination of α-KG to glutamate by branched-chain aminotransferase, yielding branched-chain ketoacids that are ultimately oxidized as succinyl -CoA in the citric acid cycle (CAC). BAIBA is a metabolite of valine in BCAA (Wagenmakers, 1992; Makowski et al., 2005; Chicco et al., 2018). It is not clear whether BCAA metabolism caused by hypoxic environment causes valine deficiency, which may be one of the reasons affecting the concentration of BAIBA in hypoxic environment. The results of this study also suggest that future research needs to further explore the influencing factors of hypoxic environment down-regulating BAIBA secretion in blood, and whether supplementation of BAIBA can further enhance the fat-reducing effect of hypoxic exercise requires further research.

In the current study, correlation analysis showed that BAIBA concentration in skeletal muscle was significantly and positively correlated with inguinal fat *PPAR α* mRNA expression level, UCP-1 mRNA expression level, and PPAR α protein expression level. Moreover, the findings showed that BAIBA concentration in blood was significantly correlated with inguinal fat *PPAR α* mRNA expression level and *UCP-1* mRNA expression level. These findings indicate that BAIBA modulates the transcriptional process of inguinal fat PPAR α and UCP-1 by regulating skeletal muscle secretion, thus regulating occurrence of white fat browning, and ultimately regulating blood lipid profile, weight loss and fat loss. Browning of white fat in obese rats occurs in visceral fat and white fat with high tissue specificity. In addition, browning of visceral fat is mainly modulated by sympathetic nerve activity and adrenaline secretion. Browning of subcutaneous fat is mainly induced by inter-tissue signaling crosstalk (Cross-talk) of cytokines, mainly muscle factors (Rodriguez et al., 2017).

## Conclusion

The finding showed that 4-weeks hypoxic exercise induced reduction in body weight and Lee’s index and improved blood lipid profile in obese rats. In addition, 4-weeks hypoxic exercise upregulated BAIBA concentration in gastrocnemius muscle and circulation, upregulated PPARα and UCP-1 expression in inguinal fat and increased white fat browning in obese rats. The findings showed that BAIBA may involves in improveing blood lipid profile lipid metabolism and white fat browning by modulating PPARα and UCP-1 expression.

## Data Availability

The raw data supporting the conclusion of this article will be made available by the authors, without undue reservation.
